# Supplementary material for: Barriers and facilitators to physical activity among Black women: A qualitative systematic review and thematic synthesis
Source: PLOS Glob Public Health. 2024 Dec 5;4(12):e0003202. doi: 10.1371/journal.pgph.0003202 (PMC11620631; doi:10.1371/journal.pgph.0003202)
Supplement: Attachment — Submitted filename: Rebuttal Letter SH.pdf [file pgph.0003202.s005.pdf]

DEPARTMENT OF KINESIOLOGY AND HEALTH EDUCATION
HEALTH BEHAVIOR AND HEALTH EDUCATION

2109 San Jacinto Blvd, Stop D3700• Austin, Texas 78712 • 512-232-6027
www.utexas.edu • slhoward@utexas.edu

Rebuttal letter:

Thank you for your initial comments on our manuscript. There were two primary concerns, which we sought to address in this revision. We hope that our response better positions this manuscript for review.

1. There was a concern that the literature review period was not specified. "Systematic and scoping reviews should provide an up-to-date overview of the research topic covered. Since the literature search was completed over a year ago, we are concerned that this study does not meet this requirement."

   Revision: We not only specified our timeframe but expanded the period of our literature search to include the years 2011-2023. Within our manuscript, we detail why we chose these specific years (page 5, lines 114-124). We added this revision to our methods section (pages 6&7, lines 150-152).

2. There was also a question regarding the systematic assessment of study quality. "In particular, we note that there is no assessment of the quality or risk of bias of the included studies. Conclusions in a systematic review or meta-analysis should be related to the quality of the included publications."

   Revision: We utilized the Critical Appraisal Skills Programme (CASP) to assess the quality of the 18 studies. This tool is described in the methods section along with our assessment (pages 7&8, lines 167-178).

Additionally, while it was not raised in the review, our expanded inclusion of articles led us to include facilitators to our use of barriers within the resulting themes. Our initial perspective reflected the deficit focus that occurred in the majority of the studies. However, the additional studies included more work on strengths – which shifted our analysis.

Your initial comments have served to significantly strengthen the manuscript and we look forward to your decision regarding peer review.
