# Supplementary material for: Barriers and facilitators to physical activity among Black women: A qualitative systematic review and thematic synthesis
Source: PLOS Glob Public Health. 2024 Dec 5;4(12):e0003202. doi: 10.1371/journal.pgph.0003202 (PMC11620631; doi:10.1371/journal.pgph.0003202)
Supplement: Attachment — Submitted filename: Editor Letter.pdf [file pgph.0003202.s006.pdf]

DEPARTMENT OF KINESIOLOGY AND HEALTH EDUCATION
HEALTH BEHAVIOR AND HEALTH EDUCATION

2109 San Jacinto Blvd, Stop D3700• Austin, Texas 78712 • 512-232-6027
www.utexas.edu • slhoward@utexas.edu

October 10, 2024

Dear Editor Tsitsi B. Masvawure, Ph.D.,

Thank you for your comments on our manuscript. There were a few areas of concern, which we sought to address in this revision. We hope that our response better positions this manuscript for acceptance.

1. There was a concern about our use of the term 'intersectionality'. "The authors do not define what they mean by the term 'intersectionality', and it is not clear how this term is being used in the paper."

   Revision: We elaborated on the definition of intersectionality and clarified how the framework is used within our study. Within our manuscript, we define intersectionality framework's origin (page 4, lines 84-93).

2. There was a request to combine tables 1 and 2. "In addition, I would like to ask the authors to incorporate Table 2 into Table 1: they should add a column on the key themes (which are currently reported in Table 2) and another column on the study objectives."

   Revision: We combined tables 1 and 2 and added the study objectives of each manuscript (pages 1-8 of figures).

3. There was a question regarding male and female athletes. "There is no discussion of Black sports icons despite their strong presence. Was this something that the studies did not ask? And if so, you need to mention that as a limitation of the reviewed studies. It would seem to me that both male and female athletes could provide good role models for PA."

   Rebuttal: Our review centers on the reported, lived experience of the participants. As such, we limited our discussion to those shared experiences and the themes that emerged. Our critique was not about these comments but, instead, centered on the approach of the research teams within the included articles. As such, we feel it is inappropriate to discuss as a weakness, the lack of other possible themes that did not arise in the studies. This is especially so as there are numerous themes that could have emerged – of which black athletes as icons is one of many that we could have discussed. As such, we did not add discussion of other, specific themes that might have emerged.

4. There was a request to add details about racial segregation. "PLOS Global Public Health is an international journal with an international readership. Hence including a little bit of details about residential segregation and White flight might be helpful especially in the part where you discuss access."

DEPARTMENT OF KINESIOLOGY AND HEALTH EDUCATION
HEALTH BEHAVIOR AND HEALTH EDUCATION

2109 San Jacinto Blvd, Stop D3700• Austin, Texas 78712 • 512-232-6027
www.utexas.edu • slhoward@utexas.edu

Revision: We added details about racial segregation to the introduction to provide context for when we discuss lack of access (page 5, lines 115-124).

5. The use of the word laziness was pointed out by one reviewer. "The researchers' understanding and labeling of themes based on the data (for example considering Doldren and Webb use of "laziness" - would you or another researcher identify that same theme in their research, or one that might have to do with time demands or competing wellness demands like connecting with and being there for family). You implicitly make this point, but it would be good to present some of this more directly and explicitly."

   Revision: The word laziness was used in the original theme, and we are bound by what is provided in the data. Within the discussion we emphasize the importance of the questions asked to elicit the response desired (page 21, lines 535-538) and the need to apply a greater level of cultural competence, with specific reference to the use of laziness as a theme (page 22, lines 545-548).

6. There was mention of the study objective being too complex. "The objective is very long, complex and incomprehensible."

   Revision: The study objective was shortened for clarity (page 7, lines 156-160).

7. There was a comment about the length of the theme sentences. "The sentence presenting the different categories of themes identified is very long and complex."

   Revision: the sentence was broken into three sentences (page 10, lines 249-253).

8. There was mention of the incorrect data analysis process. "The author should use content analysis to examine the textual corpus constituted by the set of articles selected."

   Rebuttal: Although content analysis systematically counts and categorizes explicit elements to quantify themes, a thematic synthesis is used to identify deeper, more interpretive themes that emerge from a collection of studies. A thematic synthesis is more appropriate for a systematic review as it allows for analysis and integration of findings across numerous primary studies that is more in-depth than content analysis.

9. There was a comment about internal and external validity. "The author should discuss the internal validity of the methodologies used in the articles and the external validity of these methodologies."

   Revision: We discuss issues related to internal and external validity throughout the results and discussion. For example, we discuss the lack of cultural competence and knowledge of

DEPARTMENT OF KINESIOLOGY AND HEALTH EDUCATION
HEALTH BEHAVIOR AND HEALTH EDUCATION

2109 San Jacinto Blvd, Stop D3700• Austin, Texas 78712 • 512-232-6027
www.utexas.edu • slhoward@utexas.edu

critical race theories as limitation in the existing papers (page 21, lines 538-551). This highlights issues of internal validity without labelling as such. Likewise, we discuss issues surrounding the sample of studies and their participants (page 22, lines 554-563). This highlights issues of external validity without labelling them as such. We feel adding the labels does not add to the discussion but are happy to do so in a revision if the reviewers feel strongly about the change.

10. There was a question related to differences based on region of the USA and ages of respondents. "It would be helpful if you were able to extract the data to provide age and location of the respondents. Relatedly, it would be good to know whether there were any differences in views about PA among Black women from the South versus the coasts where the influence of dominant White culture might change belief systems."

    Revision: We added this to our limitations section as this is something that should be considered in future research (page 22, lines 554-559). We also note age and location of each study in Table 1 (pages 1-8 of figures).

Your comments have served to significantly strengthen the manuscript and we look forward to your decision regarding publication.

Sincerely,

Sherron L. Howard, M.S.
Email: slhoward@utexas.edu
